# Pan-immune inflammation value as a prognostic biomarker for cancer patients treated with immune checkpoint inhibitors

**DOI:** 10.3389/fimmu.2024.1326083

**Published:** 2024-02-12

**Authors:** Tianrui Kuang, Zhendong Qiu, Kunpeng Wang, Lilong Zhang, Keshuai Dong, Weixing Wang

**Affiliations:** ^1^ Department of General Surgery, Renmin Hospital of Wuhan University, Wuhan, China; ^2^ Hubei Key Laboratory of Digestive System Disease, Wuhan, China

**Keywords:** immunotherapy, meta-analysis, prognosis, pan-immune inflammation value, tumor

## Abstract

**Background:**

Immune checkpoint inhibitors (ICIs) represent a paradigm shift in the development of cancer therapy. However, the improved efficacy of ICIs remains to be further investigated. We conducted a systematic review and meta-analysis to evaluate the pan-immunoinflammatory value (PIV) and PILE score used to predict response to ICI therapy.

**Methods:**

We searched selected databases for studies on pan-immune inflammation values and their association with outcomes of treatment with immune checkpoint inhibitors. We used hazard ratios (HRS) and 95% confidence intervals (CI) to summarize survival outcomes. All data analyses were performed using STATA 15.0.

**Results:**

7 studies comprising 982 patients were included in the meta-analysis. The pooled results showed that higher PIV was significantly associated with shorter overall survival OS (HR = 1.895, 95%CI: 1.548-2.318) and progression-free survival (PFS) (HR = 1.582, 95%CI: 1.324-1.890). Subgroup analyses also confirmed the reliability of the results.

**Conclusions:**

High PIV and PILE metrics are associated with lower survival in cancer patients receiving ICIs.

## 1 Introduction

Cancer, being the primary cause of mortality, constitutes a substantial menace to global public health as its burden continues to escalate (1). The recent emergence of monoclonal antibodies targeting immune checkpoints has fundamentally transformed the field of cancer therapy (2, 3). The success of treatment based on immune checkpoint blockade (ICB) largely relies on blocking PD-1/PD-L1 and CTLA-4 (4, 5). Immunotherapy can activate or enhance the body’s immune system, prompting it to actively target malignant tumor cells, and has demonstrated promising efficacy across multiple tumor types (6). However, the limited overall response as well as the lack of reliable biomarkers to predict patient efficacy are hampered by large (7). This underscores the pressing necessity to not only identify novel targets for immunotherapy but also easily obtainable peripheral blood indicators. These will broaden the scope of ICB-based strategies, ultimately maximizing efficacy and benefits for cancer patients.

Consequently, pan-immune inflammation value (PIV), as a novel biomarker of immune-inflammatory response, is progressively emerging as a crucial indicator for predicting the efficacy of immunotherapy and guiding individualized treatment decisions (8). Unlike traditional single-measurement biomarkers, the pan-immune inflammation value differs significantly (9). It considers the comprehensive state of the body’s immune system, along with the levels of various inflammatory signaling molecules. This integrated analysis has afforded us deeper insights into the diversity and complexity of patients’ immune statuses (10). In addition, composite prognostic scores, which incorporate many factors, have demonstrated a promising capacity for predicting prognosis in patients undergoing immunotherapy (11). The PILE score is determined by three parameters: the PIV value, the Eastern Cooperative Oncology Group Performance Status (ECOG PS), and the LDH value. Among the 120 advanced cancer patients who received anti-PD-1 or anti-PD-L1 inhibitors for various types of cancer, it was shown that a higher PILE score is linked to reduced progression-free survival (PFS) and overall survival (OS). This suggests that the PILE score has potential as a prognostic scoring system for immunotherapy (12).

This meta-analysis aimed to explore the relationship between cancer, immunotherapy, and pan-immune inflammation values. Through the integration of existing data, we sought to assess the predictive capabilities of pan-immune inflammation values in immunotherapy and their potential role in informing individualized treatment decisions. We anticipate that a comprehensive analysis of these associations will furnish robust scientific backing for refining immunotherapy strategies, enhancing treatment predictions’ precision, and ultimately advancing clinical benefits for patients.

## 2 Methods

### 2.1 Literature search

This study was conducted in compliance with the PRISMA statement guidelines (13). We conducted a systematic literature search across several electronic databases, including PubMed, EMBASE, and the Cochrane Library. The search strategy amalgamates Medical Subject Headings (MeSH) terms and pertinent keywords, such as “Immune Checkpoint Inhibitors [MeSH]”, “PD-1 Inhibitors”, “PD-L1 Inhibitors”, “CTLA-4 Inhibitors”, “Pembrolizumab”, “Nivolumab”, “Atezolizumab”, “Ipilimumab”, “pan-immune-inflammation-value”, and “ PILE “. The search is confined to studies published within a predefined time frame. Detailed search strategies are illustrated in **Supplementary Material 1**.

### 2.2 Study selection criteria

Rigorous inclusion and exclusion criteria have been systematically implemented to meticulously curate studies that adhere to high-quality standards. Eligible studies included (1) Cancer patients receiving immunotherapy; (2) Reporting of pan-immuno-inflammatory values; (3) Furthermore, these articles must report at least one of the following outcomes: overall survival (OS) or progression-free survival (PFS). We consider a diverse range of research sources, including published articles, conference abstracts, and clinical trial reports.

### 2.3 Data extraction

We meticulously extract pertinent data from the chosen studies. Key parameters include study characteristics (e.g., author, publication year), patient demographics, tumor types, treatment regimens, pan-immune inflammation value measurement methods, and clinical outcomes (e.g., OS, PFS). To ensure accuracy, multiple researchers independently conduct data extraction, with any discrepancies resolved through consensus. The PIV was determined using the equation [neutrophil count (10^3^/ml) multiplied by platelet count (10^3^/ml) multiplied by monocyte count (10^3^/ml)]. The lymphocyte count is measured in thousands per milliliter (10^3^/ml). The PILE scores were a composite score derived from the PIV, LDH level, and ECOG PS, determined by summing the separate values.

### 2.4 Quality assessment

The quality and risk of bias within individual studies are critically evaluated. Established tools such as the Newcastle-Ottawa Scale for cohort studies and the Cochrane risk of bias tool for randomized controlled trials are employed (14). Studies with inadequate methodological rigor or high risk of bias may be excluded from the final analysis to maintain the integrity of the findings.

### 2.5 Statistical analysis

Statistical analyses were performed using Stata 15.0 software. A meta-analysis employs appropriate statistical methods to quantitatively combine data across studies. Heterogeneity was evaluated using the chi-square test. The random-effects model was employed when the p-value was < 0.1 or the I2 statistic exceeded 50%, while the fixed-effects model was utilized otherwise. Publication bias can impact the comprehensiveness of the findings, and efforts are made to identify and mitigate its influence. Sensitivity analyses are performed to evaluate the robustness of the results. By systematically omitting one study at a time and re-analyzing the data, the influence of individual studies on the overall findings is assessed.

## 3 Results

### 3.1 Characteristics of studies

**Figure 1:**
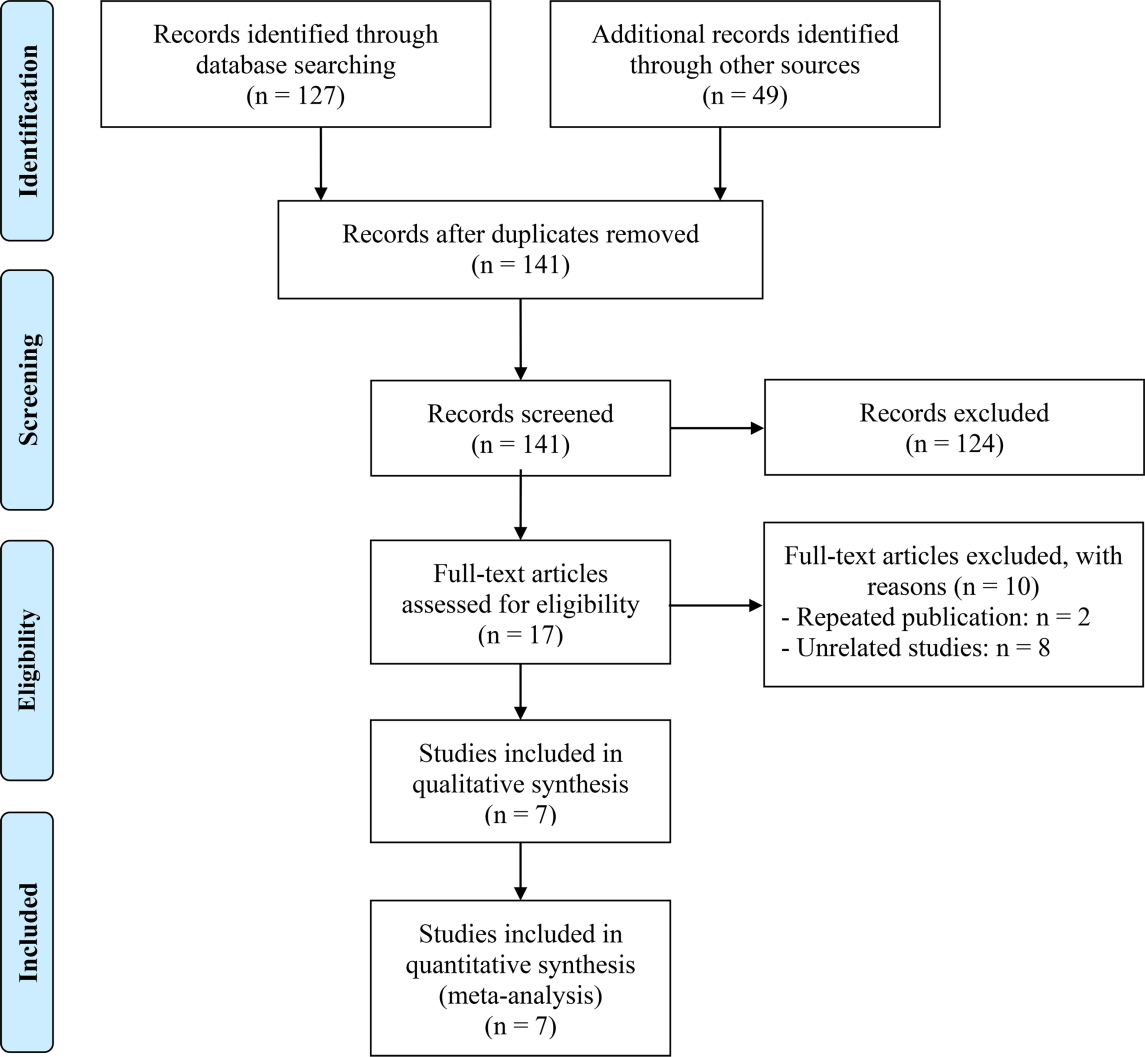
The flow diagram of identifying eligible studies.

The removal of 35 duplicate studies followed the initial search. Then, 124 articles were eliminated following a thorough examination of the titles and abstracts. The full texts of the remaining seventeen articles were subsequently evaluated in greater detail. In the end, seven articles were incorporated, and a meta-analysis was conducted on these seven investigations (12, 15–20). The flowchart for the PRISMA process is shown in **Figure 1**. **Table 1** presents the primary attributes of the studies that were incorporated. Seven studies included a total of 982 patients, and in five studies the mean age was 60 years. The NOS scores for seven articles ranged from 6 to 8, which represented a low risk of bias.

**Table 1:** Main characteristics of the studies included.

Study	Study design	Study period	Study region	ICI treatment	Cancer Type	Sample size	Age (years)	Gender (male/ female)	Outcome	NOS
Yekedüz et al. 2022 (17)	R	–	Turkey	Nivolumab	RCC	152	60(54-67)^b^	117/35	PIV (OS, PFS)	7
Corti et al. 2021 (20)	R	05/2014-06/2020	Italy	Anti-PD-(L)1 with or without anti-CTLA-4 antibodies	CRC	163	50/113^d^	90/73	PIV (OS, PFS)	7
Qi et al. 2023 (15)	P	03/2019-03/2022	China	Pembrolizumab	ESCC	51	62(39-75)^a^	44/7	PIV (PFS)	6
Sooi et al. 2022 (18)	R	01/2015-01/2022	Singapore	ICIs	NSCLC	230	63^e^	161/69	PIV (OS, PFS)	8
Mesti et al. 2022	R	01/2018-12/2020	Slovenia	Pembrolizumab, Nivolumab, Ipilimumab	Melanoma	129	66(30-85)^a^	84/53	PIV (OS, PFS)	7
Guven et al. 2021 (12)	R	06/2016-06/2020	Turkey	Anti-PD-(L)1 antibodies	Pan-cancer	120	61(54-67)^b^	86/34	PIV, PILE (OS, PFS)	7
Zeng et al. 2021 (1)	R	–	China	Atezolizumab	SCLC	53	26/27^c^	34/19	PIV, PILE (OS, PFS)	6
Zeng et al. 2021 (2)	R	01/2015-02/2021	China	Anti-PD-(L)1 antibodies	SCLC	84	37/47^c^	75/9	PIV, PILE (OS, PFS)	7

^a^medians (ranges); ^b^medians (interquartile range); ^c^≥ 65 vs. < 65; ^d^≥ 70 vs. < 70; ^e^medians; R, retrospective study; P, prospective study; ICIs, immune checkpoint inhibitors; ; PD-(L)1, programmed cell death (ligand)-1; CTLA-4, cytotoxic T-lymphocyte-associated protein 4; RCC, renal cell carcinoma; CRC, colorectal cancer; ESCC, esophageal squamous cell carcinoma; NSCLC, non-small cell lung cancer; SCLC, small cell lung cancer; OS, overall survival; PFS, progression-free survival; PIV, pan-immune-inflammation value.

### 3.2 PIV and OS

**Figure 2:**
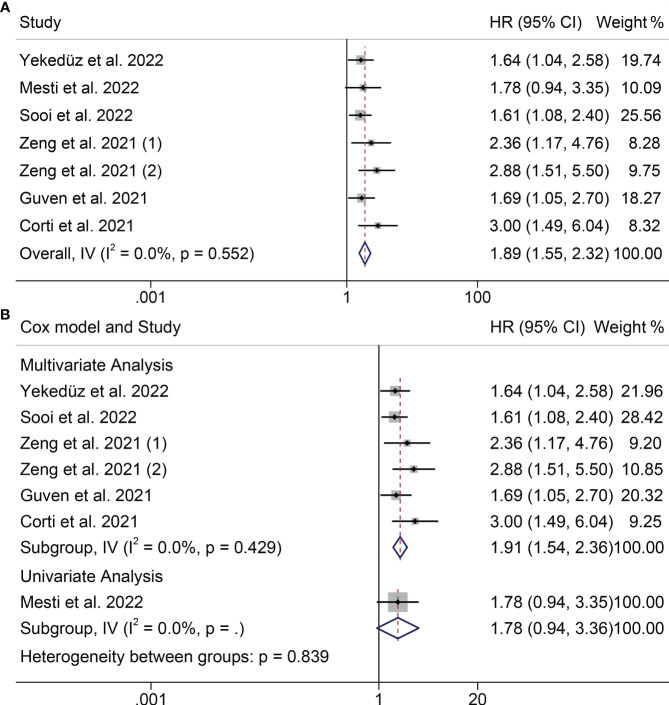
Forest plots of the relationship between PIV and OS **(A)** Meta-analysis of the overall survival. **(B)** Subgroup analysis of overall survival based on multivariate analysis and univariate analysis.

Prognostic data from six studies were used to examine the association between PIV and OS. We did not observe significant heterogeneity among the included studies (I^2 =^ 0.0%, P = 0.52, **Figure 2A**), so a fixed-effects model was used. We found that higher PIV was significantly associated with shorter OS (HR = 1.895, 95%CI: 1.548-2.318, P<0.001; **Figure 2A**).

According to its research method to subgroup analysis. In the multivariate analysis subgroups also found similar results, patients with high PIV OS shortened (HR = 1.908, 95% CI: 1.542 2.360, P < 0.001; **Figure 2B**). But subgroups according to univariate analysis result is meaningless.

### 3.3 PIV and PFS

**Figure 3:**
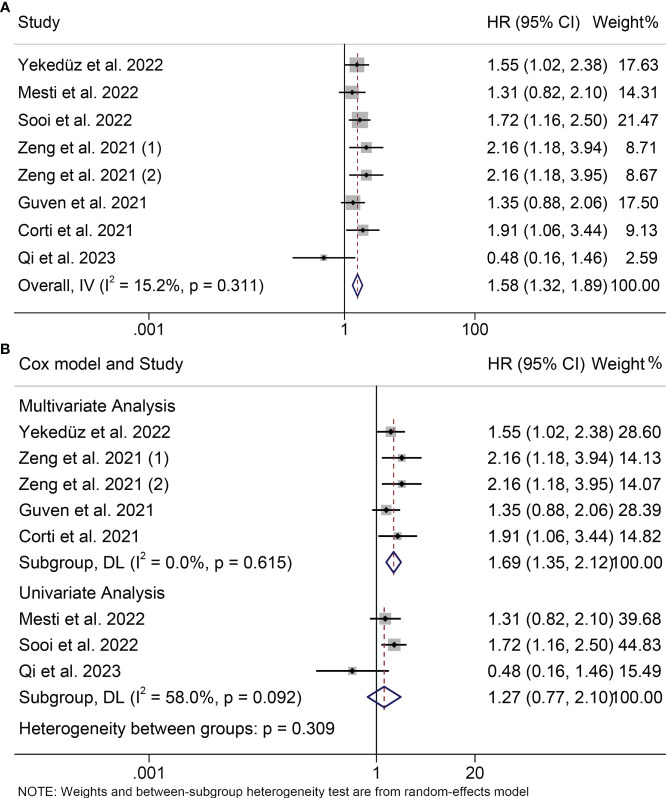
Forest plots of the relationship between PIV and PFS. **(A)** Meta-analysis of the progression-free survival. **(B)** Subgroup analysis of progression-free survival based on multivariate analysis and univariate analysis.

Seven studies with a total of 982 patients participated in the study of the relationship between PIV and PFS. Since we did not observe severe heterogeneity in these studies (I^2 =^ 15.2%, P = 0.311; **Figure 3A**), we applied a fixed model. We found that patients with high PIV levels tended to have poorer survival status (HR = 1.582, 95%CI: 1.324-1.890, P<0.001; **Figure 3A**). In the subgroup analysis, we also found that high PIV was associated with poor survival in both multivariate and univariate analyses (multivariate analyses: HR = 1.688, 95% CI: 1.346- 2.117, P < 0.001; univariate analyses: HR = 1.426, 95% CI: 1.070- 1.900, P= 0.016; **Figure 3B**).

### 3.4 PILE and PFS or OS

**Figure 4:**
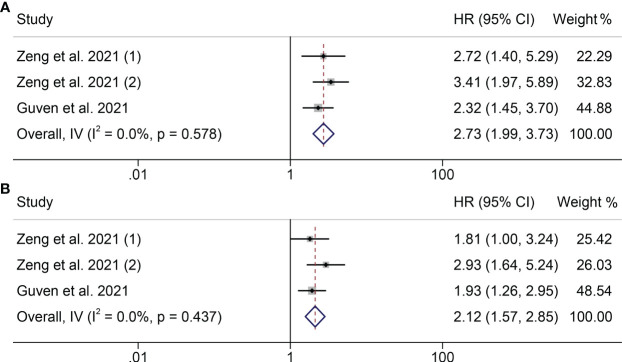
Forest plots of the relationship between PILE and ICI Efficacy in melanoma patients. **(A)** Meta-analysis of the overall survival. **(B)** Subgroup analysis of overall survival based on multivariate analysis and univariate analysis.

Next, we investigated the relationship between PILE level and clinical outcomes (OS and PFS) in cancer patients in two studies. We did not find serious heterogeneity in the studies of these two clinical outcomes, so both were fixed models used (OS: I^2 =^ 0.0%, P = 0.578; PFS: I^2 =^ 0.0%, P = 0.437; **Figure 4**). In both studies, patients with high levels of running with worse OS and PFS (OS: HR = 2.728, 95% CI: 1.993 - 3.733, P < 0.001; PFS: HR = 2.117, 95% CI: 1.574- 2.847, P < 0.001; **Figure 4**).

### 3.5 Sensitivity analysis

**Figure 5:**
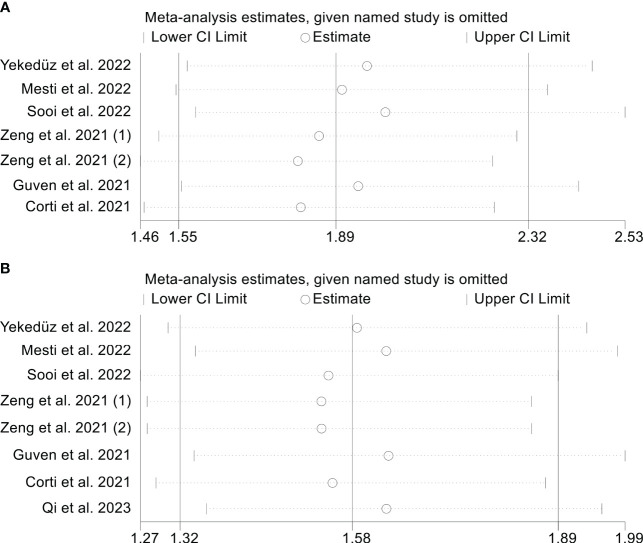
Sensitivity analysis. **(A)**. PIV and overall survival **(B)**. PIV and progression-free survival.

In order to evaluate the reliability of the results, we conducted a sensitivity analysis in which we excluded each study iteratively and assessed its effect on the overall findings. Our analysis suggests that excluding any of the studies did not have a major impact on the OS HR. Specifically, the HR estimates for OS ranged from 1.810 (95% CI: 1.464-2.239) when excluding Zeng et al., 2021 (2) to 2.003 (95% CI: 1.585-2.531) when excluding Sooi et al., (18) (**Figure 5A**). In addition, the sensitivity analysis indicated that the removal of any one study did not have a major impact on the overall results of PFS. The range of HR values varied from 1.535 (95% CI: 1.274-1.850) after excluding Zeng et al., 2021 (1) to 1.636 (95% CI: 1.345-1.990) after excluding Guven et al., (12) (**Figure 5B**).

### 3.6 Publication bias

**Figure 6:**
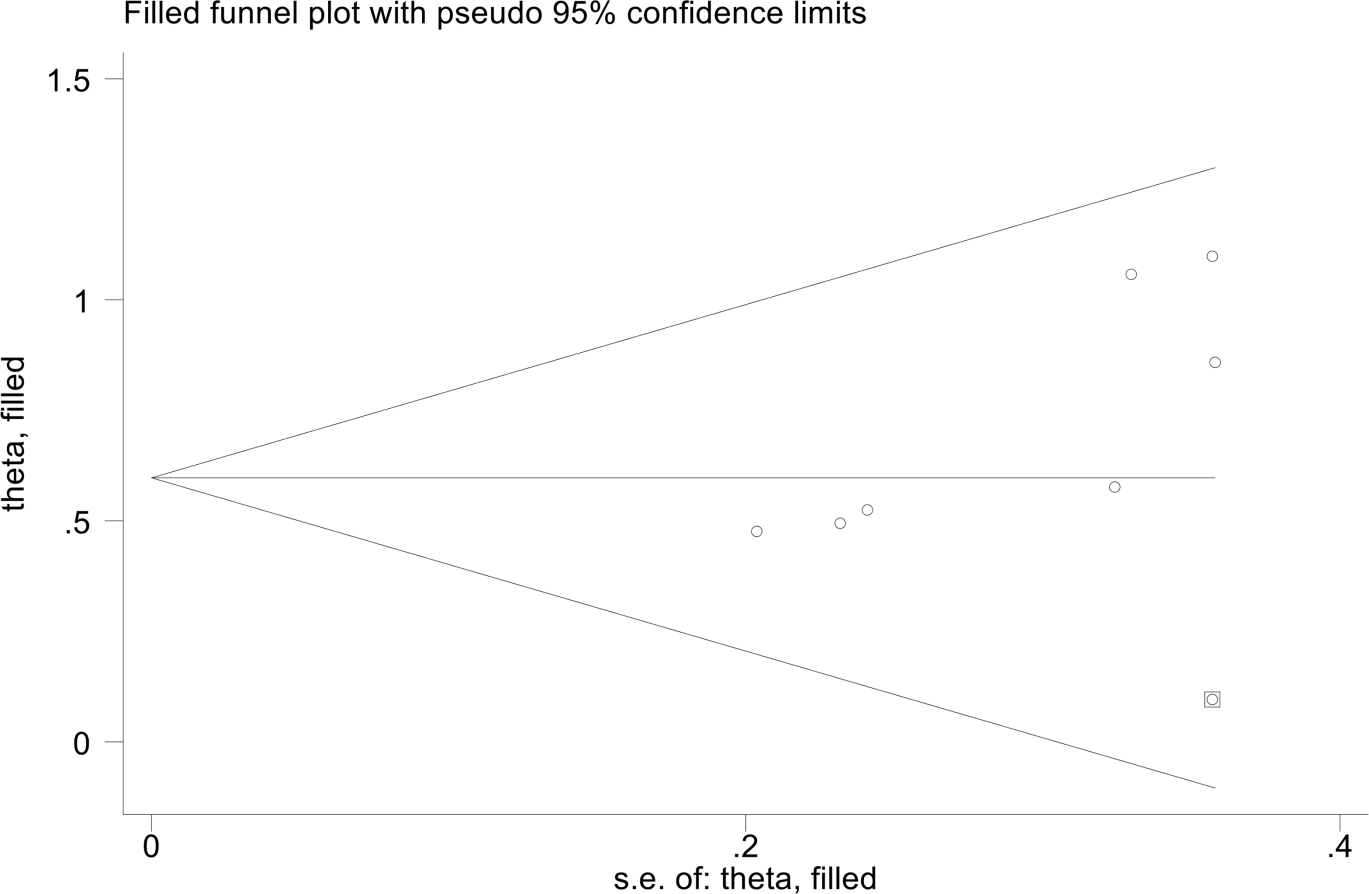
The picture of the trim-and-fill method terms of overall survival.

The results showed that PFS (Egger’s test: P = 0.455, Berg’s test: P = 1.000) no significant publication bias. However, we found that the OS is publication bias (Egger’s test: P = 0.015, Berg’s test: P = 0.016). To address this issue, we employed a trim and fill method to estimate the number of studies that might be missing in OS. The results showed no change in the combined HR when missing studies were not included (**Figure 6**).

## 4 Discussion

In this study, we aimed to explore the predictive significance of PIV and PILE in cancer patients, and the pooled data showed that higher PIV and PILE were significantly associated with shorter OS and PFS. Furthermore, the results remained stable even after conducting sensitivity and subgroup analyses. To our knowledge, this is the inaugural comprehensive meta-analysis to investigate the influence of PIV and PILE on the prognosis of cancer patients undergoing immunotherapy. As a readily available clinical indicator, assessing patients before immunotherapy can significantly enhance physicians’ ability to predict clinical outcomes and adjust treatment regimens promptly, ultimately leading to a reduction in mortality rates.

Although ICI has become a promising treatment for most cancer patients, the factors affecting its effectiveness are still unclear (21–23). Several biomarkers, including tumor mutation burden, microsatellite instability/mismatch repair deficiency, tertiary lymphoid structures, and tumor-infiltrating lymphocytes, have been suggested as potential predictors of response to ICI. However, the difficulties associated with acquiring specimens, the high cost, and the immaturity of detection technology impede the clinical application of these biomarkers. This prompted our focus on blood cells near tumors (24). These cells serve distinct roles in both promoting and inhibiting tumor growth. Neutrophils and platelets consistently promote tumor progression, whereas lymphocytes primarily drive anticancer responses in the tumor microenvironment (25, 26). The function of monocytes exhibits a heightened level of intricacy and context sensitivity. Particularly in the context of carcinogenesis, prevailing evidence suggests a prevalence of tumor-promoting effects. Studies have demonstrated a correlation between heightened secretion of pro-inflammatory cytokines and diminished monolayer confluence among tumor cells. Leveraging this paradigm, it becomes feasible to initially evaluate the effectiveness of Chimeric Antigen Receptor T-cell (CAR-T) therapy for solid tumors and gauge the potential risk of cytokine release syndrome development. Consequently, there exists an opportunity to enhance the efficacy of immunotherapy (27).

PIV is a recently devised scoring system that incorporates neutrophils, platelets, monocytes, and lymphocytes, among other immune-inflammatory cells, in peripheral blood. It has demonstrated significant utility as a prognostic biomarker in specific types of malignancies, including colon and breast cancer (8, 28, 29). It possesses a significant biological component as a prognostic factor in patients receiving ICI, reflecting systemic inflammation and immune activation. Within the spectrum of immune cell subsets implicated in cancer immune editing, tumor-associated neutrophils have been demonstrated to mechanistically enhance tumor cell migration and invasion, stimulate angiogenesis, and modulate the activity of other immune cell populations (30, 31). Filippo’s research indicates that elevated PIV levels at baseline and during early immunotherapy correlate with inferior OS and PFS outcomes (20). This measure has also been reported to predict outcomes in MSI-high metastatic colorectal cancer patients treated with immunotherapy (32). The PILE model derives its foundation from the scores of PIV, ECOG, and LDH. Due to its consideration of three indicators, it offers enhanced accuracy (11). A study demonstrated that the PILE model exhibited greater accuracy in predicting 12-week PFS and 24-week OS in the context of predicting immunotherapy efficacy (12).

It’s essential to acknowledge the limitations of this analysis. Firstly, the majority of the included studies were retrospective cohort studies, potentially affecting their statistical robustness. Secondly, the variation in the types of ICI utilized across studies introduces inconsistency. Consequently, further research, particularly well-designed, large-scale, multicenter prospective studies, is imperative to validate and enhance our findings.

## 5 Conclusion

In conclusion, cancer patients undergoing ICI treatment face diminished survival probabilities in instances characterized by elevated PIV and PILE measures. This straightforward categorization may prove beneficial in clinical application. Additional external multicenter randomized controlled trials are required to authenticate our findings on the effectiveness of the queried medication.

## Data availability statement

The original contributions presented in the study are included in the article/**Supplementary Material**. Further inquiries can be directed to the corresponding authors.

## Author contributions

TK: Writing – original draft. LZ: Software, Writing – original draft. ZQ: Writing – review & editing. KD: Writing – review & editing. WW: Writing – review & editing. KW: Writing – original draft.
