# Supplementary material for: Pan-immune inflammation value as a prognostic biomarker for cancer patients treated with immune checkpoint inhibitors
Source: Front Immunol. 2024 Feb 12;15:1326083. doi: 10.3389/fimmu.2024.1326083 (PMC10895004; doi:10.3389/fimmu.2024.1326083)
Supplement: Supplementary file 1 [file Table_1.docx]

Table S1 Detailed search strategy.

((((((((((((((((((((((((((((((((((((((((((((((((Camrelizumab) OR (Sintilimab)) OR (Tislelizumab)) OR (Toripalimab)) OR (Envafolimab)) OR (Immune Checkpoint Inhibitors)) OR (Checkpoint Inhibitors, Immune)) OR (Immune Checkpoint Inhibitor)) OR (Checkpoint Inhibitor, Immune)) OR (Immune Checkpoint Blockers)) OR (Checkpoint Blockers, Immune)) OR (Immune Checkpoint Blockade)) OR (Checkpoint Blockade, Immune)) OR (Immune Checkpoint Inhibition)) OR (Checkpoint Inhibition, Immune)) OR (PD-L1 Inhibitors)) OR (PD L1 Inhibitors)) OR (PD-L1 Inhibitor)) OR (PD L1 Inhibitor)) OR (Programmed Death-Ligand 1 Inhibitors)) OR (Programmed Death Ligand 1 Inhibitors)) OR (PD-1-PD-L1 Blockade)) OR (Blockade, PD-1-PD-L1)) OR (PD 1 PD L1 Blockade)) OR (CTLA-4 Inhibitors)) OR (CTLA 4 Inhibitors)) OR (CTLA-4 Inhibitor)) OR (CTLA 4 Inhibitor)) OR (Cytotoxic T-Lymphocyte-Associated Protein 4 Inhibitors)) OR (Cytotoxic T Lymphocyte Associated Protein 4 Inhibitors)) OR (Cytotoxic T-Lymphocyte-Associated Protein 4 Inhibitor)) OR (Cytotoxic T Lymphocyte Associated Protein 4 Inhibitor)) OR (PD-1 Inhibitors)) OR (PD-1 Inhibitor)) OR (PD 1 Inhibitors)) OR (Inhibitor, PD-1)) OR (PD 1 Inhibitor)) OR (Programmed Cell Death Protein 1 Inhibitor)) OR (Programmed Cell Death Protein 1 Inhibitors)) OR (Pembrolizumab)) OR (Nivolumab)) OR (Atezolizumab)) OR (Ipilimumab)) OR (Avelumab)) OR (Tremelimumab)) OR (Durvalumab)) OR (Cemiplimab)) OR (Immune Checkpoint Inhibitors[MeSH Terms])) AND ((((pan-immune-inflammation-value) OR (pan-Immune-inflammatory value)) OR (PIV)) OR (PILE))
